# Development and validation of a diabetic retinopathy risk prediction model for middle-aged patients with type 2 diabetes mellitus

**DOI:** 10.3389/fendo.2023.1132036

**Published:** 2023-03-15

**Authors:** Gao-Xiang Wang, Xin-Yu Hu, Heng-Xia Zhao, Hui-Lin Li, Shu-Fang Chu, De-Liang Liu

**Affiliations:** ^1^ Department of Endocrinology, Shenzhen Traditional Chinese Medicine Hospital Affiliated to Nanjing University of Chinese Medicine, Shenzhen, Guangdong, China; ^2^ Department of Endocrinology, Shenzhen Traditional Chinese Medicine Hospital, Shenzhen, Guangdong, China; ^3^ Department of Endocrinology, The Fourth Clinical Medical College of Guangzhou University of Chinese Medicine, Shenzhen, Guangdong, China

**Keywords:** prediction model, nomogram, diabetic retinopathy, middle-aged, type 2 diabetes mellitus

## Abstract

**Objectives:**

The study aims to establish a predictive nomogram of diabetic retinopathy(DR) for the middle-aged population with type 2 diabetes mellitus (T2DM).

**Methods:**

This retrospective study screened 931 patients with T2DM between 30 and 59 years of age from the 2011-2018 National Health and Nutrition Examination Survey database. The development group comprised 704 participants from the 2011-2016 survey, and the validation group included 227 participants from the 2017-2018 survey. The least absolute shrinkage and selection operator regression model was used to determine the best predictive variables. The logistic regression analysis built three models: the full model, the multiple fractional polynomial (MFP) model, and the stepwise (stepAIC) selected model. Then we decided optimal model based on the receiver operating characteristic curve (ROC). ROC, calibration curve, Hosmer-Lemeshow test, and decision curve analysis (DCA) were used to validate and assess the model. An online dynamic nomogram prediction tool was also constructed.

**Results:**

The MFP model was selected to be the final model, including gender, the use of insulin, duration of diabetes, urinary albumin-to-creatinine ratio, and serum phosphorus. The AUC was 0.709 in the development set and 0.704 in the validation set. According to the ROC, calibration curves, and Hosmer-Lemeshow test, the nomogram demonstrated good coherence. The nomogram was clinically helpful, according to DCA.

**Conclusion:**

This study established and validated a predictive model for DR in the mid-life T2DM population, which can assist clinicians quickly determining who is prone to develop DR.

## Introduction

Diabetic retinopathy (DR) is a usual microvascular complication of type 2 diabetes mellitus (T2DM), one of the major reasons for blindness (1). According to The Global Burden of Disease Study, DR was the only cause of age-standardized vision loss to increase over the past three decades (2). Over 103.12 million adults worldwide were diagnosed with DR in 2020, and with the prevalence of diabetes increasing at an alarming rate, it is estimated that the world DR population will grow by 55.6%(57.4 million) between 2020 and 2045 (3). A prevalence-based cost-of-illness model estimates that Indonesia will spend $8.9 billion on the healthcare of DR in 2025 (4). As DR is often asymptomatic until the later, even more, severe stages, early diagnosis, and intervention are essential and more cost-effective for public health and healthcare costs (5–7).

DR prevalence has been discussed in some studies in different age groups of T2DM. The ADVANCE Collaborative group (8) has reported that the course of diabetes is independently related to the risk of microvascular complications, and diabetes duration has a more significant impact on younger people than on older people. Middleton et al. (9) have found that DR seems more susceptible in people diagnosed with T2DM in middle age (or with a younger present age), and the odds of DR decreased with increasing age at diagnosis. They considered this difference to be caused by reducing insulin-like growth factor 1 and growth hormone with increasing age. DR is more likely to occur in the middle-aged population after diagnosis of T2DM than in the elderly (10), so a more targeted prediction model and intervention strategy are needed.

Several prediction models have been applied to the identification and diagnosis of DR (11–13). However, these prediction models were constructed for almost all age groups. They have shortcomings in predicting the development of DR in different age groups. This will limit their ability to stratify individual patients according to risk level and select the optimal treatment. To our knowledge, there is a lack of predictive models developed separately for the middle-aged population. We suggested that developing a separate DR prediction model for the middle-aged age group and narrowing the prediction model orientation may be more important for applying the model for early identification and prevention of DR. We developed a model predicting the development of DR in middle-aged people with T2DM based on data from the National Health and Nutrition Examination Survey (NHANES), which may provide more personalized screening and treatment options for middle-aged T2DM patients.

## Materials and methods

### Study design and participants

NHANES is a study program to evaluate US adults’ and kids’ health and nutritional condition. They sampled about 5,000 nationally representative persons with a multistage, graded, clustered sampling approach every year (14).

**Figure 1:**
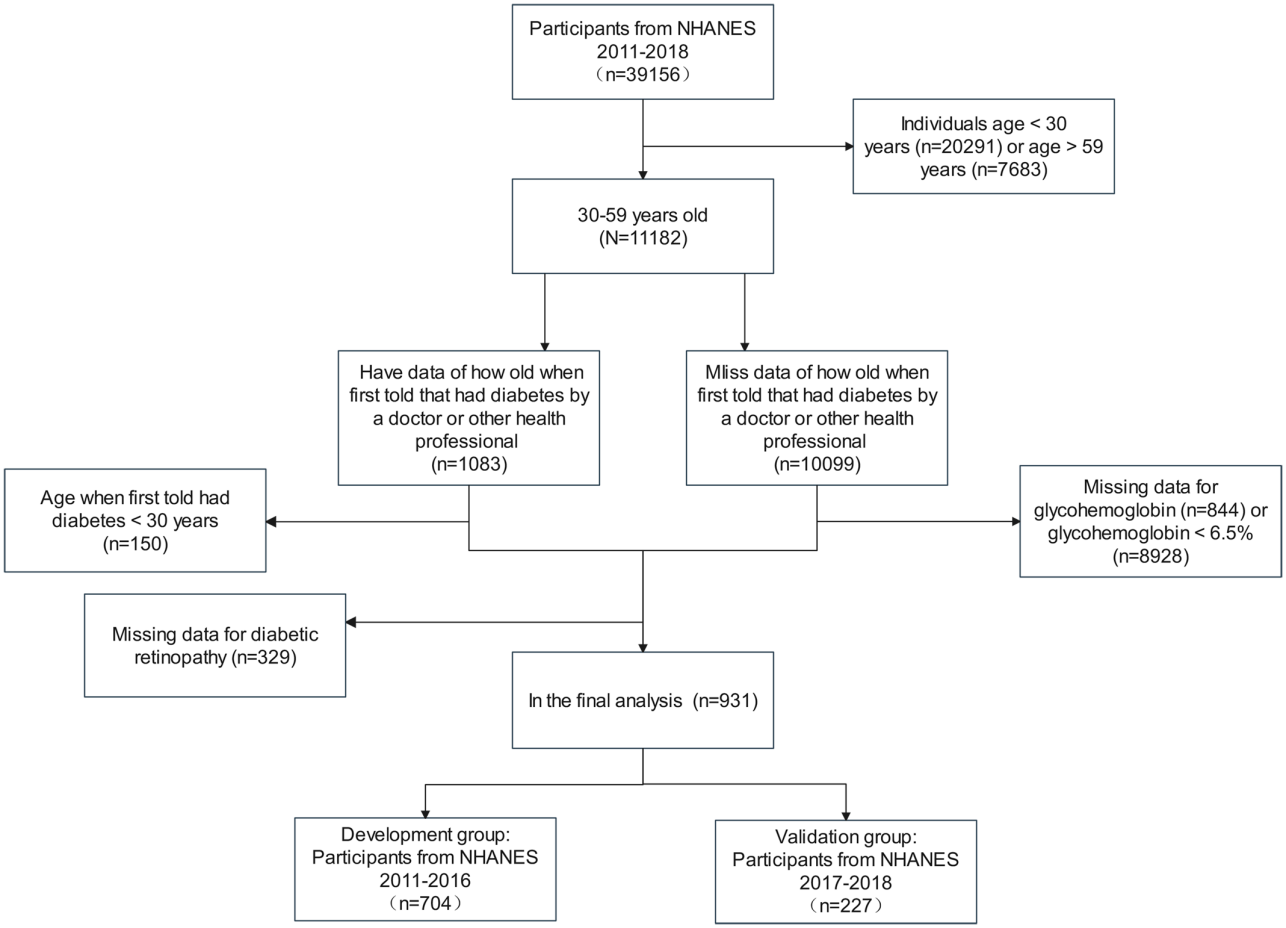
Flow chart of the development and validation groups.

We included 39,156 participants in this study from the NHANES 2011 to 2018. According to the guideline from the American Diabetes Association (15), patients with T2DM were defined as follows: (1) participants who a doctor told them that they had diabetes with a diagnosis age ≥30 years; (2) participants who didn’t self-report diabetes diagnosis with HbA1c ≥6.5%. We excluded data for participants <30 years (n=20,291) and >59 years (n=7,683) to obtain 11,182 cases in the age group of 30-59 years. Then, participants were separated into two groups depending on whether or not they had data of how old they were first told by a professional that they had diabetes, with data in the first group (n=1,083) and miss data in the second group (n=10,099). The first group excluded participants who were younger than 30 years old when they were first told they had diabetes and those who had no data for DR, resulting in 604 participants. The second group excluded patients with missing glycohemoglobin data and glycohemoglobin<6.5%, resulting in 327 cases. The two data groups were combined to get the final population included in the analysis for this study. The population from 2011 to 2016 was used to establish the development cohort, and the population from 2017 to 2018 was adopted as an external validation cohort. **Figure 1** illustrates the detailed selection operation.

### Ethics statement

Each participator provided written informed agreement before inclusion in the NHANES database, which was examined and allowed by the National Center for Health Statistics Ethics Review Board. Anonymously processing the data makes it available to the public. The researchers then can transform the data into a form suitable for analysis following privacy-preserving. Based on the study’s data usage guidelines, all data will be analyzed statistically, and all studies will comply with all relevant laws and standards.

### Potential predictors

We selected some potential predictors which might affect DR progress based on current relevant research and clinical experience (16–18), including age, gender, diabetes duration, HbA1C, use of insulin, use of hypoglycemic pills, hypertension, weak failing kidneys, body mass index (BMI), waist circumference, alkaline phosphatase, alanine aminotransferase (ALT), aspartate aminotransferase (AST), serum calcium, serum phosphorus, serum potassium, serum uric acid, total cholesterol, triglyceride, serum calcium, serum iron, blood urea nitrogen, serum albumin, serum creatinine, urinary albumin-to-creatinine ratio(UACR). The information on hypertension and renal failure came from the questionnaires.

### Statistical analysis

R statistical software version 3.6.3 and EmpowerStats version 2.0 were used to conduct the statistical analysis for this study. Data for normally distributed was displayed as the mean ± standard deviation, and a two independent samples *t*-test was performed to analyze differences between groups. The categorical variables were described with proportion, which was tested using the chi-square test.

In linear regression mode, least absolute shrinkage and selection operator (LASSO) regression analysis is used for shrinkage and variable option. Firstly, we used the development set data and analyzed the data using the LASSO regression method. LASSO regression analysis was used to determine the appropriate and effective risk predictors for T2DM patients with DR, and 7 independent variables were selected according to lambda.min. Then, we built three models based on the logistic regression analysis: the full model, the multiple fractional polynomial (MFP) model, and the stepwise (stepAIC) selected model. We used the odds ratio and *P*-value with 95% confidence interval (CI) to describe the features. At the same time, according to the comparison of the area under the receiver operating characteristic (ROC) curve of each model in the development set and the validation set, the model with the most significant area under the curve (AUC) was selected. The model’s consistency was evaluated based on the calibration curve and the Hosmer-Lemeshow test. The clinical effectiveness of the model was assessed using decision curve analysis (DCA). All statistical analyses were two-sided, with an alpha of 0.05 as the significance grade. Finally, according to the model, we established the nomogram and online dynamic nomogram prediction tool.

## Results

### Baseline characteristics

According to the prespecified exclusion and inclusion criteria, 931 participants were enrolled in our research, including 704 in the development group and 227 in the validation group. Baseline characteristics like demographic, biochemical indexes, physical examination findings, duration of diabetes, and the use of medications are shown in **Table 1**.

**Table 1:** Characteristics of the study sample.

	Development group (n=704)	Validation group (n=227)	P value
**Age (years)**	50.12 ± 6.74	50.10 ± 6.74	0.957
Gender (%)			0.591
**male**	50.41	52.41	
**female**	49.59	47.59	
**Duration of diabetes (years)**	6.61 ± 5.44	6.79 ± 5.60	0.653
**Glycohemoglobin (%)**	7.67 ± 1.96	7.53 ± 1.74	0.346
Taking insulin now(%)			0.621
No	76.81	78.36	
Yes	23.19	21.64	
Take diabetic pills now (%)			0.174
No	28.47	23.96	
Yes	71.53	76.04	
High blood pressure (%)			0.949
No	36.55	36.78	
Yes	63.45	63.22	
Had weak failing kidneys (%)			0.076
No	94.36	97.21	
Yes	5.64	2.79	
**Body mass index (kg/m^2^)**	34.62 ± 8.13	36.03 ± 8.03	0.021
**Waist circumference (cm)**	113.48 ± 16.96	115.65 ± 16.36	0.084
**Alkaline phosphatase (u/L)**	73.27 ± 23.27	83.87 ± 29.03	<0.001
**Alanine aminotransferase (u/L)**	73.27 ± 23.27	83.87 ± 29.03	<0.001
**Aspartate aminotransferase (u/L)**	28.15 ± 25.90	24.68 ± 17.89	0.054
**Serum calcium (mmol/L)**	2.35 ± 0.09	2.32 ± 0.11	<0.001
**Serum phosphorus (mmol/L)**	1.23 ± 0.18	1.14 ± 0.19	<0.001
**Serum potassium (mmol/L)**	4.03 ± 0.33	4.12 ± 0.41	<0.001
**Serum uric acid (umol/L)**	329.25 ± 90.31	323.61 ± 84.66	0.395
**Total cholesterol (mmol/L)**	4.89 ± 1.21	4.83 ± 1.15	0.553
**Triglyceride (mmol/L)**	2.53 ± 3.00	2.40 ± 2.06	0.554
**Serum iron (umol/L)**	13.89 ± 5.67	14.89 ± 6.95	0.027
**Blood urea nitrogen (mmol/L)**	5.11 ± 2.17	5.29 ± 2.15	0.242
**Serum albumin (g/L)**	41.89 ± 3.42	39.69 ± 3.44	<0.001
**Serum creatinine (umol/L)**	81.01 ± 65.60	76.17 ± 55.82	0.305
**Urinary albumin creatinine ratio (mg/g)**	125.75 ± 598.05	166.02 ± 840.45	0.421
Diabetic retinopathy (%)			0.423
No	83.57	81.32	
Yes	16.43	18.68	

Continuous variables are displayed as mean ± standard deviation. Based on a linear regression model, the *P-*value was calculated. The categorical variables were described with proportion, *P-*value was calculated by the chi-square test.

### Risk factors in the development group

**Figure 2:**
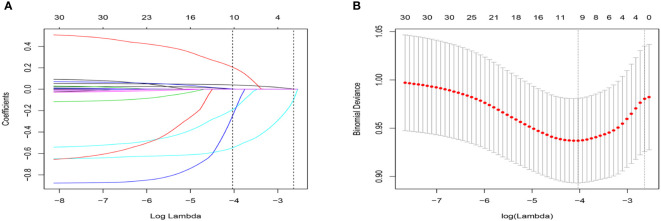
Selection of variables using the LASSO regression model. **(A)** The coefficient profile was plotted against the log (lambda) sequence. **(B)** The plot of partial likelihood deviance (binomial deviance) versus log (lambda) was performed.

We included 24 associated characteristic variables in LASSO regression analysis (**Figures 2A, B**) and selected 7 non-zero potential predictors from the LASSO regression analysis results based on the data of the development group. These predictors included gender, taking insulin now, weak failing kidneys, duration of diabetes, UACR, blood urea nitrogen, and serum phosphorus.

### Prediction model development

**Figure 3:**
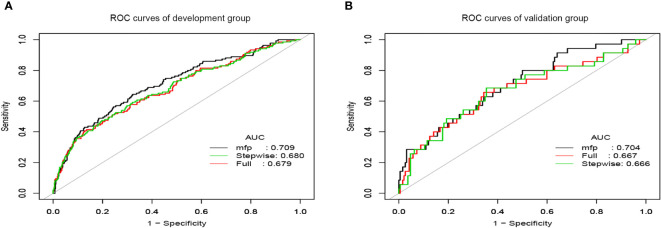
The ROC curves of prediction models. **(A)** ROC curves of the development group. **(B)** ROC curves of the validation group.

To construct the prediction model, we performed the following steps. Firstly, we combined all 7 potential predictors selected by LASSO regression analysis into a multivariable model using multivariate logistic regression, which built the full model. Then, the stepwise backward regression selection method was used to fit the stepwise model based on Akaike’s Information Criterion (AIC). Since the multicollinearity of the predictors, we constructed the multiple fractional polynomial (MFP) model. The details of the three models are shown in **Table 2**. Finally, ROC curves were plotted for all three models, and the AUC of these models was compared (**Figure 3**). We chose the MFP model to construct the nomogram according to the results.

**Table 2:** Logistic regression analysis for risk factors in three models.

Model	Estimate	Std error	Odds ratio	95%CI.low	95%CI.upp	P-value
Model 1						
(Intercept)	-0.6306	0.6663	0.5323	0.1442	1.9648	0.3439
Gender=female	-0.3878	0.2093	0.6786	0.4503	1.0227	0.0639
Taking insulin now=yes	0.7185	0.2347	2.0513	1.2949	3.2495	0.0022
Weak failing kidneys=yes	0.4099	0.3718	1.5066	0.7269	3.1225	0.2703
Duration of diabetes	0.052	0.0172	1.0534	1.0185	1.0895	0.0025
Urinary albumin creatinine ratio	0.0004	0.0002	1.0004	1	1.0007	0.0253
Blood urea nitrogen	0.0367	0.0447	1.0374	0.9505	1.1323	0.4108
Serum phosphorus	-1.2588	0.581	0.284	0.0909	0.887	0.0303
Model 2						
(Intercept)	-0.6154	0.6668	0.5404	0.1463	1.9967	0.356
Gender= female	-0.4129	0.2071	0.6617	0.4409	0.9931	0.0462
Taking insulin now=yes	0.7216	0.2345	2.0577	1.2994	3.2584	0.0021
Weak failing kidneys=yes	0.5119	0.3479	1.6684	0.8437	3.2991	0.1412
Duration of diabetes	0.0533	0.0171	1.0548	1.0199	1.0908	0.0019
Urinary albumin creatinine ratio	0.0004	0.0002	1.0004	1.0001	1.0007	0.0194
Serum phosphorus	-1.1235	0.5558	0.3251	0.1094	0.9665	0.0432
Model 3						
(Intercept)	0.089	0.7097	1.0931	0.272	4.3933	0.9002
Gender=female	-0.4145	0.2061	0.6607	0.4411	0.9896	0.0444
Taking insulin now=yes	0.7175	0.2307	2.0492	1.3039	3.2206	0.0019
Duration of diabetes/10	0.5111	0.1718	1.6672	1.1905	2.3347	0.0029
(Urinary albumin creatinine ratio/100)^-0.5	-0.3199	0.0734	0.7262	0.6288	0.8387	<0.0001
Serum phosphorus	-0.9159	0.5467	0.4001	0.137	1.1683	0.0939

Model 1: Full model:Risk of Diabetic retinopathy=-0.63058 -0.38775*(Gender=female) +0.05199*Duration of diabetes +0.00037*Urinary albumin creatinine ratio +0.40987*(Weak failing kidneys=yes) +0.71848*(Taking insulin now=yes) +0.03673*Blood urea nitrogen-1.25877*Serum phosphorus.

Model 2: Stepwise (stepAIC) selected model: Risk of Diabetic retinopathy=-0.61541 -0.41290*(Gender=female) +0.05331*Duration of diabetes +0.00039*Urinary albumin creatinine ratio +0.51186*(Weak failing kidneys=yes) +0.72158*(Taking insulin now=yes) -1.12349*Serum phosphorus.

Model 3:Multiple Fractional Polynomial mode:Risk of Diabetic retinopathy=0.08903 +0.71746*(Taking insulin now=Yes) +0.51112*(Duration of diabetes/10)-0.31991*((Urinary albumin creatinine ratio/100)^-0.5) -0.91593*Serum phosphorus -0.41447*(Gender= Female).

### Development of nomogram

**Figure 4:**
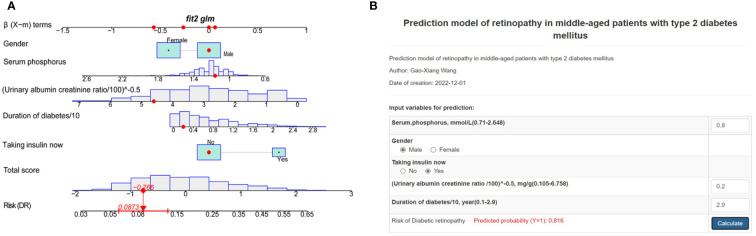
Risk nomogram development. **(A)** An example of the dynamic nomogram. **(B)** An example of the online dynamic nomogram tool.

According to the MFP model, 5 independent predictors were introduced to establish a DR risk nomogram (**Figure 4A**). To make it more convenient for T2DM patients to predict the progress of DR, we created an online dynamic nomogram tool (http://www.empowerstats.net/pmodel/?m=22793_GaoXiangWangredictionmodelofretinopathyinmiddleagedpatientswithtype2diabetesmellitus). In the online tool, doctors can calculate the risk of DR in middle-aged T2DM patients based on the specific values of each indicator (**Figure 4B**).

### Assessment of predictive nomogram

We applied the ROC curve to test the discrimination of the model (**Figures 3A, B**). In the development group, the AUC was 0.709(95%CI:0.659-0.759) for the MFP model, 0.680 (95% CI:0.627-0.733) for the stepwise model, and 0.679 (95%CI:0.627-0.732) for the full model (**Figure 3A**). And in the validation group, the AUC was 0.704(95%CI:0.611-0.798) for the MFP model, 0.667(95% CI:0.567-0.774) for the full model, and 0.666 (95%CI:0.560-0.773)for the stepwise model (**Figure 3B**).

**Figure 5:**
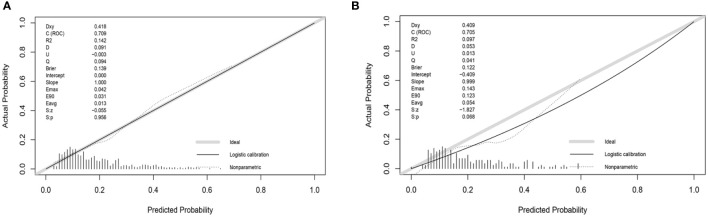
Calibration curve of the risk nomogram. **(A)** Calibration curve of development group. **(B)** Calibration curve of validation group.

To check the consistency of this model, calibration curves and the Hosmer-Lemeshow test were used. As shown in **Figure 5**, the calibration curves of the model in both the development and validation sets were plotted. The horizontal axis stands for the predicted DR risk, the vertical coordinates represent the actual diagnosed DR risk, and the gray diagonal line stands for the perfect prediction of the ideal nomogram. Nomogram performance is shown by the solid line, where the closer to the diagonal gray line suggests greater predictive performance. According to the calibration curves, the nomogram displayed good coherence. In addition, there was no significant difference between the validation and development groups when we used the Hosmer-Lemeshow test to test the model calibration degree. (*P*=0.42 in the development group, *P*=0.52 in the validation group).

**Figure 6:**
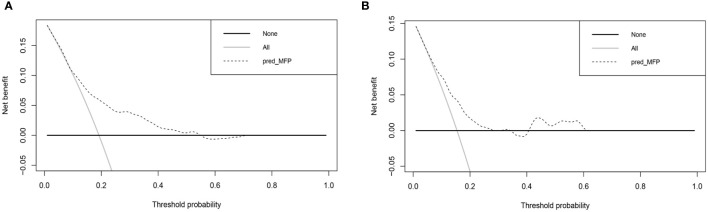
Decision curve analysis for the nomogram. **(A)** Decision curve analysis of development group. **(B)** Decision curve analysis of validation group.

**Figure 6** shows the results of DCA curves for development and validation groups. The dashed line stood for the model, the gray line showed the net benefit when all patients with DR, and the black line represented the net benefit when no patients with DR. The region of the model curve between the “black line” and the “gray line” represented the model’s clinical applicability. If the dashed line is above the black and gray lines, we can assume that the dashed value of the period can benefit.

## Discussion

Nomogram is a useful and reliable forecasting tool that can produce individual probabilities of endpoint events by combining different variables and quantifying the risk individually (19). In the risk predictor analysis of this study, gender, use of insulin, renal failure, duration of diabetes, UACR, blood urea nitrogen, and serum phosphorus were related to the risk of DR in midlife patients with T2DM. Based on this, we used statistical analysis to screen five of these variables to construct and validate a novel DR risk predictive tool for middle-aged patients with T2DM. The model showed that being male, taking insulin now, longer duration of diabetes, higher UACR, and lower serum phosphorus were critical factors in determining the risk of DR in patients with T2DM, which has the same part of risk factors as those reported in previous studies (10, 20). To make it more convenient for physicians to provide early individualized intervention for middle-aged T2DM patients, we have built an online, free prediction tool. According to Anne et al. (21), the AUC value of 0.7 or higher is considered acceptable or good for model discrimination. Our model presented good discrimination and calibration ability, offering a personalized prediction of DR incidence.

For patients with T2DM, the course of the disease is an unchangeable risk factor. Unlike T1DM, disease duration has a more significant impact on patients with T2DM combined with DR (22). Compared to older T2DM patients, midlife patients have a longer survival time and will be exposed to the increased risk of complications associated with a longer disease course. Elevated blood glucose and lipid metabolism disorders in T2DM patients caused pathological reactions, such as oxidative stress and inflammatory response (23, 24), which were considered an important pathogenesis of DR (25–27), Longer disease duration means a sustained state of inflammation for a longer period, which raises the risk of DR. As reported by Singh et al. (28), DR prevalence was five times higher in patients with a disease duration of >15 years than it was in patients with an illness duration of < 5 years. According to Sun et al. (29), the duration of diabetes is strongly correlated with the risk of DR. In middle-aged patients with T2DM. It is critical to diagnose and intervene in the early stages of DR progression to minimize the risks that come with a longer disease course.

Gender has been discovered in some studies to be relevant to the risk of developing diabetes-related complications (30, 31). Middle-aged men were significantly more likely to have T2DM, indicating that gender factors are involved to some extent in the pathogenesis of T2DM and its complications in the middle-aged population (32, 33). Several studies have revealed sexual dimorphism in fat distribution, inflammatory signaling pathway activation, and T2DM risk (34–37). Middle-aged T2DM patients have demonstrated gender differences in disease progression and pathogenesis, and our model suggested that being male is significantly associated with DR in the middle-aged population. Studies based on national databases from the UK and Finland found that the male sex is an independent risk factor for advanced DR in T2DM and a risk predictor for disease progression (38, 39). Maric-Bilkan et al. (40) concluded that age-related differences in hormone levels, glycemic control, duration of diabetes, and ethnic background could explain the reported gender differences in DR risk. Although the pathological mechanisms of gender influence on DR progression are unclear now, the significantly different prevalence between gender implied different individualized care measures. Prevention strategies targeting modifiable risk factors are critical for the middle-aged T2DM population.

Since its first clinical use in 1922, exogenous insulin has become a widely used hypoglycemic drug for many forms of diabetic patients worldwide (41). Our results found that mid-aged T2DM patients on insulin therapy were at greater risk of developing DR. A meta-analysis based on seven cohort studies has shown a significant association between the use of insulin and the risk of DR (42). A systematic review conducted by Song et al. (43) discovered that insulin therapy was remarkably correlated with an increased prevalence of any DR. The correlation between insulin therapy and DR demonstrated in various studies indicated that clinicians need to be more cautious when applying insulin therapy to patients at high risk of developing DR. Besides when dealing with patients on long-term insulin therapy, DR should be detected more carefully.

UACR is a clinically used indicator of renal function and a marker of endothelial dysfunction and may affect the microvasculature of the kidney and retina. Wang et al. (44) found that UACR, in addition to being an important marker of chronic kidney disease, was also closely related to the progression of DR. A 10-year prospective follow-up study confirmed that both UACR and estimated glomerular filtration rate (eGFR) were significant risk factors for DR, but UACR had a more significant association than eGFR (45), which is consistent with our result. The current studies found that high UACR is linked to changes in retinal vascular geometry, that patients with high UACR appear to be potentially predisposed to systemic vascular endothelial cell disease, and glycemic control may not affect the inherent biological risk of developing microvascular complications (46, 47). For this reason, UACR may be a favorable and easily accessible biochemical indicator for predicting DR.

Although the prediction model developed in this research is meaningful for the early prevention and treatment of the middle-aged T2DM population, there are still some limitations. First, the population included in this study was the general middle-aged US population. Due to the differences in lifestyle and eating habits, the DR prediction nomogram may be limited in its generalization to other national people. Second, all patient data in this study were obtained from the NHANES database. Although we used data from different periods for validation, multicenter clinical validation is needed to assess the efficacy of the nomogram. Third, we could not refine our DR study according to whether it was proliferative due to the lack of data limitations of DR staging in the NHANES database data.

The study developed a new web-based nomogram for predicting DR prevalence in middle-aged T2DM patients. After internal and external validation, the nomogram demonstrated good predictive performance. The line chart includes 5 common clinical characteristics of gender, serum phosphorus, UACR, duration of diabetes, and use of insulin. This nomogram enabled early to identify the high-risk groups of DR in middle-aged T2DM patients and helped to develop an aggressive individualized prevention and treatment strategy to reduce the prevalence and slow down the progression of DR. More clinical prospective and multicenter trials are needed to confirm our nomogram.

## Data availability statement

This study uses data from a free and open public database, which can be found here: www.cdc.gov/nchs/nhanes/.

## Ethics statement

Ethical approval was not provided for this study on human participants because each participator provided written informed agreement before inclusion in the NHANES database, which was examined and allowed by the National Center for Health Statistics Ethics Review Board. Anonymously processing the data makes it available to the public. The researchers then can transform the data into a form suitable for analysis following privacy-preserving. Based on the study’s data usage guidelines, all data will be analyzed statistically, and all studies will comply with all relevant laws and standards. Written informed consent for participation was not required for this study in accordance with the national legislation and the institutional requirements.

## Author contributions

G-XW and X-YH contributed equally to this study. All the authors contributed to the article and approved the final version.

## References

[1] Lin K Hsih W Lin Y Wen C Chang T. Update in the epidemiology, risk factors, screening, and treatment of diabetic retinopathy. J Diabetes Investig (2021) 12:1322–5. doi: 10.1111/jdi.13480 PMC8354492 33316144

[2] Steinmetz JD Bourne RRA Briant PS Flaxman SR Taylor HRB Jonas JB. Causes of blindness and vision impairment in 2020 and trends over 30 years, and prevalence of avoidable blindness in relation to VISION 2020: the right to sight: an analysis for the global burden of disease study. Lancet Global Health (2021) 9:e144–60. doi: 10.1016/S2214-109X(20)30489-7 PMC7820391 33275949

[3] Teo ZL Tham Y-C Yu M Chee ML Rim TH Cheung N. Global prevalence of diabetic retinopathy and projection of burden through 2045. Ophthalmology (2021) 128:1580–91. doi: 10.1016/j.ophtha.2021.04.027 33940045

[4] Sasongko MB Wardhana FS Febryanto GA Agni AN Supanji S Indrayanti SR. The estimated healthcare cost of diabetic retinopathy in Indonesia and its projection for 2025. Br J Ophthalmol (2020) 104:487–92. doi: 10.1136/bjophthalmol-2019-313997 31285276

[5] Wong TY Sun J Kawasaki R Ruamviboonsuk P Gupta N Lansingh VC. Guidelines on diabetic eye care. Ophthalmology (2018) 125:1608–22. doi: 10.1016/j.ophtha.2018.04.007 29776671

[6] Vujosevic S Aldington SJ Silva P Hernández C Scanlon P Peto T. Screening for diabetic retinopathy: New perspectives and challenges. Lancet Diabetes Endocrinol (2020) 8:337–47. doi: 10.1016/S2213-8587(19)30411-5 32113513

[7] Siegel KR Ali MK Zhou X Ng BP Jawanda S Proia K. Cost-effectiveness of interventions to manage diabetes: Has the evidence changed since 2008? Diabetes Care (2020) 43:1557–92. doi: 10.2337/dci20-0017 33534729

[8] for the ADVANCE Collaborative group Zoungas S Woodward M Li Q ME C Hamet P. Impact of age, age at diagnosis and duration of diabetes on the risk of macrovascular and microvascular complications and death in type 2 diabetes. Diabetologia (2014) 57:2465–74. doi: 10.1007/s00125-014-3369-7 25226881

[9] Middleton TL Constantino MI Molyneaux L D’Souza M Twigg SM Wu T. Young-onset type 2 diabetes and younger current age: Increased susceptibility to retinopathy in contrast to other complications. Diabetes Med (2020) 37:991–9. doi: 10.1111/dme.14238 PMC7317898 31968129

[10] Liu Y Yang J Tao L Lv H Jiang X Zhang M. Risk factors of diabetic retinopathy and sight-threatening diabetic retinopathy: A cross-sectional study of 13 473 patients with type 2 diabetes mellitus in mainland China. BMJ Open (2017) 7:e016280. doi: 10.1136/bmjopen-2017-016280 PMC5588996 28864696

[11] Li J Guo C Wang T Xu Y Peng F Zhao S. Interpretable machine learning-derived nomogram model for early detection of diabetic retinopathy in type 2 diabetes mellitus: A widely targeted metabolomics study. Nutr Diabetes (2022) 12:36. doi: 10.1038/s41387-022-00216-0 35931671 PMC9355962

[12] Chen X Xie Q Zhang X Lv Q Liu X Rao H. Nomogram prediction model for diabetic retinopathy development in type 2 diabetes mellitus patients: A retrospective cohort study. J Diabetes Res (2021) 2021:1–8. doi: 10.1155/2021/3825155 PMC8478593 34595241

[13] Shan Y Wang Q Zhang Y Tong X Pu S Xu Y. High remnant cholesterol level is relevant to diabetic retinopathy in type 2 diabetes mellitus. Lipids Health Dis (2022) 21:12. doi: 10.1186/s12944-021-01621-7 35057797 PMC8772129

[14] Xu Z McClure S Appel L. Dietary cholesterol intake and sources among U.S adults: Results from national health and nutrition examination surveys (NHANES), 2001–2014. Nutrients (2018) 10:771. doi: 10.3390/nu10060771 29903993 PMC6024549

[15] American Diabetes Association Professional Practice Committee. 2. classification and diagnosis of diabetes: Standards of medical care in diabetes–2022. Diabetes Care (2022) 45:S83–96. doi: 10.2337/dc22-S006 34964868

[16] Yang H Xia M Liu Z Xing Y Zhao W Li Y. Nomogram for prediction of diabetic retinopathy in patients with type 2 diabetes mellitus: A retrospective study. J Diabetes its Complications (2022) 36:108313. doi: 10.1016/j.jdiacomp.2022.108313 36183450

[17] Li Y Li C Zhao S Yin Y Zhang X Wang K. Nomogram for prediction of diabetic retinopathy among type 2 diabetes population in xinjiang, China. DMSO (2022) 15:1077–89. doi: 10.2147/DMSO.S354611 PMC8999722 35418766

[18] Mo R Shi R Hu Y Hu F. Nomogram-based prediction of the risk of diabetic retinopathy: A retrospective study. J Diabetes Res (2020) 2020:1–9. doi: 10.1155/2020/7261047 PMC7298262 32587869

[19] Balachandran VP Gonen M Smith JJ DeMatteo RP. Nomograms in oncology: More than meets the eye. Lancet Oncol (2015) 16:e173–80. doi: 10.1016/S1470-2045(14)71116-7 PMC4465353 25846097

[20] Yang J Jiang S. Development and validation of a model that predicts the risk of diabetic retinopathy in type 2 diabetes mellitus patients. Acta Diabetol (2022) 60:43–51. doi: 10.1007/s00592-022-01973-1 36163520

[21] de Hond AAH Steyerberg EW van Calster B. Interpreting area under the receiver operating characteristic curve. Lancet Digit Health (2022) 4:e853–5. doi: 10.1016/S2589-7500(22)00188-1 36270955

[22] He M Long P Chen T Li K Wei D Zhang Y. ALDH2/SIRT1 contributes to type 1 and type 2 diabetes-induced retinopathy through depressing oxidative stress. Oxid Med Cell Longev (2021) 2021:1641717. doi: 10.1155/2021/1641717 34725563 PMC8557042

[23] Kang Q Yang C. Oxidative stress and diabetic retinopathy: Molecular mechanisms, pathogenetic role and therapeutic implications. Redox Biol (2020) 37:101799. doi: 10.1016/j.redox.2020.101799 33248932 PMC7767789

[24] Busik JV. Lipid metabolism dysregulation in diabetic retinopathy. J Lipid Res (2021) 62:100017. doi: 10.1194/jlr.TR120000981 33581416 PMC7892987

[25] Catrina S-B Zheng X. Hypoxia and hypoxia-inducible factors in diabetes and its complications. Diabetologia (2021) 64:709–16. doi: 10.1007/s00125-021-05380-z PMC7940280 33496820

[26] Forrester JV Kuffova L Delibegovic M. The role of inflammation in diabetic retinopathy. Front Immunol (2020) 11:583687. doi: 10.3389/fimmu.2020.583687 33240272 PMC7677305

[27] Augustine J Troendle EP Barabas P McAleese CA Friedel T Stitt AW. The role of lipoxidation in the pathogenesis of diabetic retinopathy. Front Endocrinol (2021) 11:621938. doi: 10.3389/fendo.2020.621938 PMC7935543 33679605

[28] Singh HV Das S Deka DC Kalita IR. Prevalence of diabetic retinopathy in self-reported diabetics among various ethnic groups and associated risk factors in north-East India: A hospital-based study. Indian J Ophthalmol (2021) 69:3132–7. doi: 10.4103/ijo.IJO_1144_21 PMC8725127 34708755

[29] Sun Q Jing Y Zhang B Gu T Meng R Sun J. The risk factors for diabetic retinopathy in a Chinese population: A cross-sectional study. J Diabetes Res (2021) 2021:5340453. doi: 10.1155/2021/5340453 33575359 PMC7861953

[30] Peters SAE Woodward M. Sex differences in the burden and complications of diabetes. Curr Diabetes Rep (2018) 18:33. doi: 10.1007/s11892-018-1005-5 PMC5906520 29671082

[31] Huebschmann AG Huxley RR Kohrt WM Zeitler P Regensteiner JG Reusch JEB. Sex differences in the burden of type 2 diabetes and cardiovascular risk across the life course. Diabetologia (2019) 62:1761–72. doi: 10.1007/s00125-019-4939-5 PMC7008947 31451872

[32] Li J Ni J Wu Y Zhang H Liu J Tu J. Sex differences in the prevalence, awareness, treatment, and control of diabetes mellitus among adults aged 45 years and older in rural areas of northern China: A cross-sectional, population-based study. Front Endocrinol (2019) 10:147. doi: 10.3389/fendo.2019.00147 PMC6426742 30923514

[33] Sattar N. Gender aspects in type 2 diabetes mellitus and cardiometabolic risk. Best Pract Res Clin Endocrinol Metab (2013) 27:501–7. doi: 10.1016/j.beem.2013.05.006 24054927

[34] Henstridge DC Abildgaard J Lindegaard B Febbraio MA. Metabolic control and sex: A focus on inflammatory-linked mediators. Br J Pharmacol (2019) 176:4193–207. doi: 10.1111/bph.14642 PMC6877797 30820935

[35] Winkler TW Justice AE Graff M Barata L Feitosa MF Chu S. The influence of age and sex on genetic associations with adult body size and shape: A Large-scale genome-wide interaction study. PloS Genet (2015) 11:e1005378. doi: 10.1371/journal.pgen.1005378 26426971 PMC4591371

[36] Pulit SL Karaderi T Lindgren CM. Sexual dimorphisms in genetic loci linked to body fat distribution. Bioscience Rep (2017) 37:BSR20160184. doi: 10.1042/BSR20160184 PMC5291139 28073971

[37] de Ritter R de Jong M Vos RC van der Kallen CJH Sep SJS Woodward M. Sex differences in the risk of vascular disease associated with diabetes. Biol Sex Differ (2020) 11:1. doi: 10.1186/s13293-019-0277-z 31900228 PMC6942348

[38] Looker HC Nyangoma SO Cromie D Olson JA Leese GP Black M. Diabetic retinopathy at diagnosis of type 2 diabetes in Scotland. Diabetologia (2012) 55:2335–42. doi: 10.1007/s00125-012-2596-z PMC3411303 22688348

[39] Kostev K Rathmann W. Diabetic retinopathy at diagnosis of type 2 diabetes in the UK: A database analysis. Diabetologia (2013) 56:109–11. doi: 10.1007/s00125-012-2742-7 23052061

[40] Maric-Bilkan C. Sex differences in micro- and macro-vascular complications of diabetes mellitus. Clin Sci (2017) 131:833–46. doi: 10.1042/CS20160998 28424377

[41] Mathieu C Martens P-J Vangoitsenhoven R. One hundred years of insulin therapy. Nat Rev Endocrinol (2021) 17:715–25. doi: 10.1038/s41574-021-00542-w 34404937

[42] Zhao C Wang W Xu D Li H Li M Wang F. Insulin and risk of diabetic retinopathy in patients with type 2 diabetes mellitus: Data from a meta-analysis of seven cohort studies. Diagn Pathol (2014) 9:130. doi: 10.1186/1746-1596-9-130 24972631 PMC4227060

[43] Song P Yu J Chan KY Theodoratou E Rudan I. Prevalence, risk factors and burden of diabetic retinopathy in China: A systematic review and meta-analysis. J Global Health (2018) 8:10803. doi: 10.7189/jogh.08.010803 PMC5997368 29899983

[44] Wang J Xin X Luo W Wang R Wang X Si S. Anemia and diabetic kidney disease had joint effect on diabetic retinopathy among patients with type 2 diabetes. Invest Ophthalmol Vis Sci (2020) 61:25. doi: 10.1167/iovs.61.14.25 PMC7757636 33351059

[45] Romero-Aroca P Baget-Bernaldiz M Navarro-Gil R Moreno-Ribas A Valls-Mateu A Sagarra-Alamo R. Glomerular filtration rate and/or ratio of urine albumin to creatinine as markers for diabetic retinopathy: A ten-year follow-up study. J Diabetes Res (2018) 2018:5637130. doi: 10.1155/2018/5637130 29682579 PMC5846354

[46] Benitez-Aguirre PZ Marcovecchio ML Chiesa ST Craig ME Wong TY Davis EA. Urinary albumin/creatinine ratio tertiles predict risk of diabetic retinopathy progression: A natural history study from the adolescent cardio-renal intervention trial (AdDIT) observational cohort. Diabetologia (2022) 65:872–8. doi: 10.1007/s00125-022-05661-1 PMC8960571 35182158

[47] Benitez-Aguirre PZ Wong TY Craig ME Davis EA Cotterill A Couper JJ. The adolescent cardio-renal intervention trial (AdDIT): Retinal vascular geometry and renal function in adolescents with type 1 diabetes. Diabetologia (2018) 61:968–76. doi: 10.1007/s00125-017-4538-2 PMC6447498 29396691

